# Lead contamination in human milk affects infants’ language trajectory: results from a prospective cohort study

**DOI:** 10.3389/fpubh.2024.1450570

**Published:** 2024-08-13

**Authors:** Nathalia Ferrazzo Naspolini, Pedro A. R. Vanzele, Pedro Tótolo, Paulo Alfonso Schüroff, Daniel Fatori, Santos Alves Vicentini Neto, Cristiane Barata-Silva, Lisia Maria Gobbo dos Santos, André Fujita, Maria Rita Passos-Bueno, Patricia C. B. Beltrão-Braga, Alline C. Campos, André C. P. L. F. Carvalho, Guilherme V. Polanczyk, Josino Costa Moreira, Carla R. Taddei

**Affiliations:** ^1^School of Arts, Sciences and Humanity, University of São Paulo, São Paulo, Brazil; ^2^Department of Clinical and Toxicological Analyses, School of Pharmaceutical Sciences, University of São Paulo, São Paulo, Brazil; ^3^Institute of Mathematics and Statistics, University of São Paulo, São Paulo, Brazil; ^4^Department of Psychiatry, Faculdade de Medicina FMUSP, University of São Paulo, São Paulo, Brazil; ^5^Laboratorio de Psicopatologia e Terapeutica Psiquiatrica LIM-23, Instituto de Psiquiatria, Hospital das Clinicas HCFMUSP, Faculdade de Medicina, Universidade de São Paulo, São Paulo, Brazil; ^6^Department of Chemistry, National Institute for Quality Control in Health (INCQS), Oswaldo Cruz Foundation (INCQS/Fiocruz), Rio de Janeiro, Brazil; ^7^Division of Network AI Statistics, Medical Institute of Bioregulation, Kyushu University, Fukuoka, Japan; ^8^Department of Genetics and Evolutionary Biology, Institute of Biosciences, University of São Paulo, São Paulo, Brazil; ^9^Department of Microbiology, Institute of Biomedical Sciences, University of São Paulo, São Paulo, Brazil; ^10^Institut Pasteur de São Paulo, São Paulo, Brazil; ^11^Department of Pharmacology, Ribeirao Preto Medical School, University of São Paulo, São Paulo, Brazil; ^12^Department of Applied Mathematics and Statistics, Institute of Mathematics and Computer Sciences, University of São Paulo, Sao Carlos, Brazil

**Keywords:** environmental exposure, human milk, infant development, lead exposure, language trajectory

## Abstract

Infants growing up in low- and middle-income countries are at increased risk of suffering adverse childhood experiences, including exposure to environmental pollution and lack of cognitive stimulation. In this study, we aimed to examine the levels of metals in the human milk of women living in São Paulo City, Brazil, and determine the effects on infants’ neurodevelopment. For such, a total of 185 human milk samples were analyzed for arsenic (As), lead (Pb), mercury (Hg), and cadmium (Cd) using inductively coupled plasma mass spectrometry (ICP-MS). We applied the Bayley scales of infant and toddler development Third Edition (Bayley-III) to assess developmental milestones. In our analysis, we found a mean (standard deviation) concentration of As in human milk equal to 2.76 (4.09) μg L^−1^, followed by Pb 2.09 (5.36) and Hg 1.96 (6.68). Cd was not detected. We observed that infants exposed to Pb presented language trajectories lower than non-exposed infants (β = −0.413; 95% CI -0.653, −0.173) after adjustment for infant age, maternal education, socioeconomic status, infant sex, and sample weights. Our results report As, Pb, and Hg contamination in human milk, and that infant exposure to Pb decreased infants’ language development. These results evidence maternal-child environmental exposure and its detrimental impact on infants’ health.

## 1 Introduction

Infants in low- and middle-income countries (LMICs) have the highest risk of being exposed to environmental pollution (1). According to the Lancet Commission on Pollution and Health (2017), there is a significant inequity in pollution-related deaths, with the highest burden in LMICs (2). The Commission reported that pollution was responsible for approximately 9 million deaths (16% of all deaths globally) in 2015, and more than 90% of these deaths occurred in LMICs (2). Pollution includes the contamination of air by fine particulate matter (PM2.5); the contamination of the ocean by mercury, nitrogen, plastic, and petroleum waste; and the poisoning of the land by lead, mercury, pesticides, industrial chemicals, and electronic waste, among others (3).

Exposure to environmental pollutants can be particularly dangerous for infants, as their metabolic capacity and biochemical pathways are immature, and their organs and systems are still developing (4). These contaminants represent a significant risk for exclusively breastfed infants since human milk can contain contaminants, due to environmental exposure being their only source of nutrition during the initial months of life (5). Toxic exposures before birth or in early postnatal life can lead to short-term death in infancy and childhood, as well as chronic non-communicable diseases (NCDs), including neurobehavioral disorders (6), that may manifest at any point throughout the human lifespan (6, 7).

Epidemiological evidence has highlighted the risks associated with environmental exposures that may result in alterations to brain development (8–11). Understanding the impact of exposures during the first 1,000 days can shed light on brain development in LMIC populations. Furthermore, metal exposure and its association with neurodevelopment are poorly understood and require further confirmation in different human populations. This study aimed to investigate the association between infants’ exposure to arsenic (As), lead (Pb), mercury (Hg), and cadmium (Cd) via human milk and adverse neurodevelopmental outcomes.

## 2 Materials and methods

We conducted a longitudinal analysis on the Germina cohort (12), a population-based infant cohort from the metropolitan area of São Paulo. One hundred and eighty-five healthy mother-infant dyads provided human milk samples at 3 months of age. Infants’ neurodevelopment was assessed at 3, 5–9, and 10–16 months using the Bayley scales of infant and toddler development 3rd Edition (Bayley-III). The Bayley-III consists of a series of tasks and behavioral observations, including the following domains: cognitive, language, motor, and social–emotional development. The Bayley-III was previously translated and adapted to Brazilian Portuguese, and we used the composite scores in this study (13).

Ethical approvals were obtained from the Ethics Committee for the Analysis of Research Projects (CAPPESq) and the National Council of Ethics in Research (ref.: CAAE 49671221.2.0000.0068). Following the Declaration of Helsinki, all mothers provided written informed consent before completing any study measure.

We analyzed the levels of As, Pb, Hg, and Cd in the samples using acid digestion with 65% nitric acid (w/v) and inductively coupled plasma mass spectrometry (ICP-MS) model NexION 300D, manufactured by PerkinElmer, United States. The ICP-MS system was equipped with a concentric nebulizer (Meinhard), cyclonic glass nebulizer chamber, cone, skimmer, and nickel hyper-skimmer technique at the National Institute of Quality Control in Health Laboratory (INCQS) (14). The limit of detection (LOD) was determined by analyzing 10 independent blank solutions and calculated according to the National Institute of Metrology, Standardization, and Industrial Quality (INMETRO) guidance document for a 95% confidence level (15). The accuracy and precision of the method were assessed through a recovery study. Their acceptance criteria typically range from 80 to 120% of the certified value, with a maximum relative standard deviation (% RSD) below 30% (15, 16). The metal’s limits of quantification (LOQ) were: Pb 0.015 μg/dL, Hg 0.007 μg L^−1^, Cd 0.002 μg L^−1^, As 0.003 μg L^−1^. For values below the LOQ, we imputed the limit of detection (LOD) divided by two, as suggested for right-skewed data distribution (17). Since the imputation rate was higher than 50%, infants were categorized as exposed or non-exposed for further analysis It is essential to note that the group of infants categorized as “non-exposed to Pb” were not exposed to detectable levels of this metal in human milk samples according to the technique used. However, exposure below LOD and from other sources cannot be ruled out. To compare differences between infant groups we used chi-square test for categorical variables and t-test for r continuous variables. The metal levels of the exposed infants are presented as the mean (SD).

We used a linear mixed-effects model [*nlme* R package (18)] with repeated measures to assess the relationship between longitudinal language scores and Pb exposure, with the presence and absence of the interaction term between infant age and Pb exposure. The adjustment process incorporated key socio-economic variables, maternal ethnicity and education, socioeconomic status score, infant age, and sex. The socioeconomic status score was defined based on Brazil’s Criteria for Economic Classification. We constructed pseudo-weights (19) to improve the representativeness of our sample relative to the general population. All analyses were conducted in R.^1^

## 3 Results

We evaluated the levels of metals in human milk samples (*n* = 185). The highest detection rate was for As (38.6%), followed by Hg (23.9%) and Pb (22.8%). The mean (SD) concentration was 2.76 (4.09) μg/L, 1.96 (6.68), 2.09 (5.36), respectively. Cd was not detected (Table 1).

**Table 1:** Levels of metals in human milk.

Metals	*n*	Detection *n* (% > LOQ)	Mean (SD)	Min/Max
Arsenic	185	71 (38.6%)	2.76 (4.09)	0.10–34.55
Mercury	185	44 (23.9%)	1.96 (6.68)	0.04–54.41
Lead	185	42 (22.8%)	2.09 (5.36)	0.15–40.90
Cadmium	185	0	-	-

Germina Study, São Paulo, Brazil, 2021–2022. Metals units are μg L^−1^; Limits of quantification: Pb 1 μg L^−1^; Hg 0.3 μg L^−1^; Cd 0.08 μg L^−1^; As 0.06 μg L^−1^; LOQ, Limit of Quantification.

Further, we analyzed the associations of As, Pb, Hg, and Pb exposure and the Bayley-III cognitive and language composite scores across time points, and only Pb showed a significant effect in the Bayley language composite trajectory (Supplementary Table 1). Therefore, the subsequent analyses are regarding Pb and the language Bayley Scale. Maternal and infant characteristics according to Pb exposure are described in Table 2. No significant differences were observed for total family income, maternal education, infant sex, gestational age, and socio-economic between the infant groups. Infants exposed to Pb had significantly lower (mean[sd]) performance in the language Bayley Scale at 10–16 months of age (97.47 [13.36]) compared to non-exposed infants (102.96 [11.53]).

**Table 2:** Maternal and infants’ characteristics.

	Total	Exposed	Non-exposed	*p*-value^*^
Total family income (US dollar)	2,378.9 (2,476.7)	2,339.6 (1,971.7)	2,390.4 (2,611.5)	0.912
Maternal educational attainment				0.763
>College degree	125 (79.62)	29 (76.3%)	103 (78.6%)	
<College degree	32 (20.38)	9 (23.7%)	28 (21.4%)	
Infant sex				0.310
Female	81 (51.59)	23 (60.5%)	67 (51.1%)	
Male	76 (48.41)	15 (39.5%)	64 (48.9%)	
Gestational age (weeks)	39.02 (1.02)	38.95 (1.11)	39.05 (0.99)	0.601
Socioeconomic status score	37.15 (8.73)	37.13 (7.74)	37.16 (9.02)	0.986
**Bayley language composite score**				
At 3 months of age	104.30 (8.30)	102.76 (8.55)	104.74 (8.21)	0.197
At 5–9 months of age	103.01 (9.17)	101.66 (10.20)	103.40 (8.85)	0.303
At 10–16 months of age	101.73 (12.14)	97.47 (13.36)	102.96 (11.53)	**<0.001**
Lead (μg/L)	2.09 (5.36)	0.15 (0.00)	8.67 (8.43)	**<0.001**

Germina Study—São Paulo, Brazil, 2021–2022. Mean (SD); *N* (%). ^*^The chi-square test was used for categorical variables and the t-test for continuous variables. Bold values represent statistically significant results.

**Figure 1:**
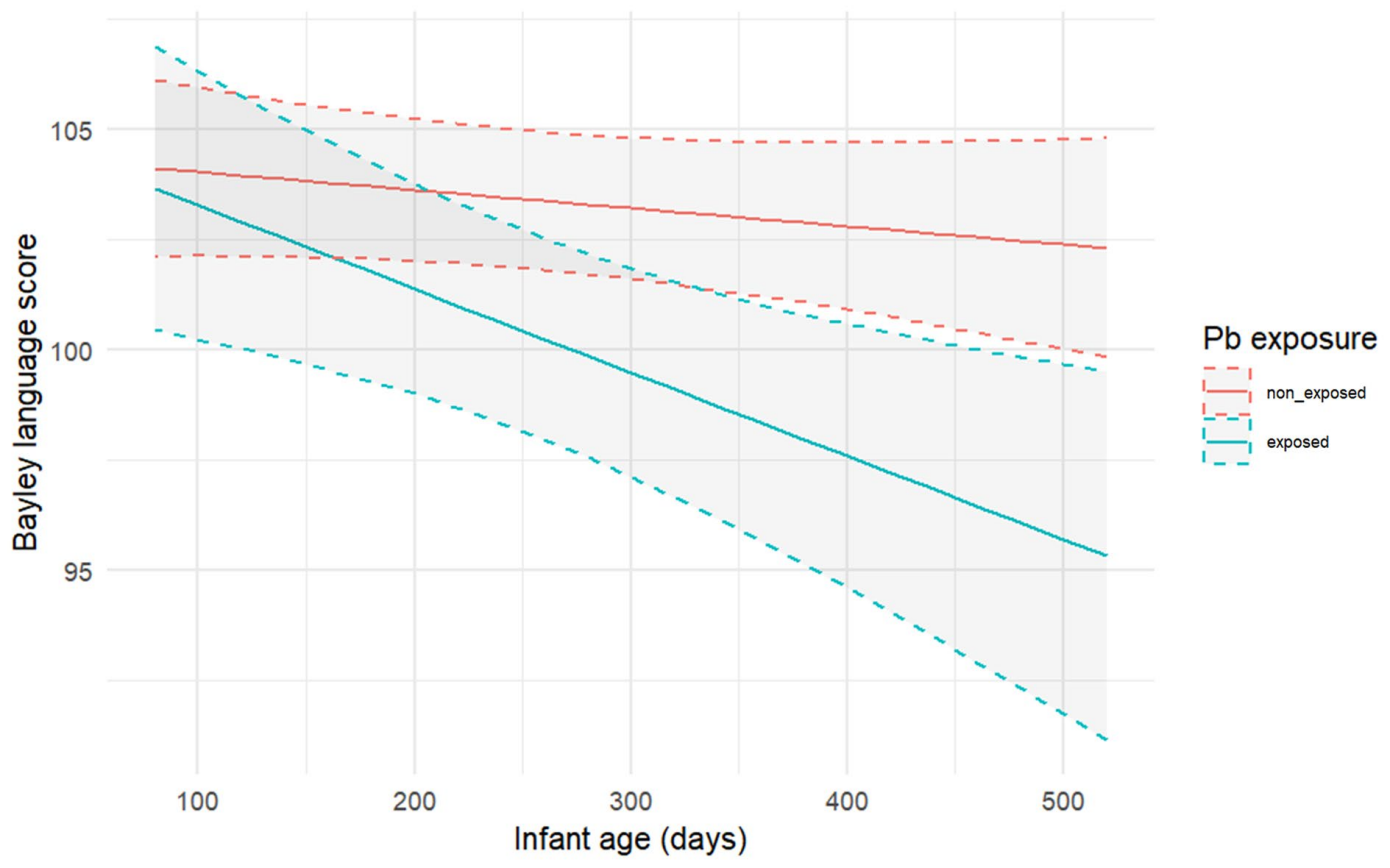
Infants exposed to Pb had lower language trajectories compared to non-exposed infants. X-axis are infant age in days and Y-axis are infants language Bayley trajectory stratified by Pb exposure status.

Table 3 shows the regression coefficients for infants’ language trajectory predictors. The joint effect of Pb exposure and infant age resulted in a decreased language trajectory only among the Pb exposed infant group (β = −0.413; 95% CI = −0.653, −0.173), adjusting for infant age, socioeconomic status, maternal education, infant sex, and sample weights (Figure 1). Socioeconomic status was also a significant predictor of language trajectory (β = 0.129; 95% CI = 0.002, 0.255).

**Table 3:** Regression coefficients of language trajectories predictors.

	β	95% CI	*p*-value
Infant age (days)	−0.142	−0.338 **–** 0.053	0.155
Socioeconomic status score	0.129	**0.002**–**0.255**	**0.048**
Maternal educational attainment	−0.634	−3.383 – 2.114	0.651
Infant sex	0.539	−1.420 – 2.498	0.590
Pb exposure*Infant age	**−0.413**	**−0.653 – −0.173**	**<0.001**

Germina Study—São Paulo, Brazil, 2021–2022. Bold values represent statistically significant results.

## 4 Discussion

In this study, we measured the levels of metals in human milk and their association with infant language development during the first 2 years of life. We found that about one-third of the human milk samples had detectable Pb, As, and Hg levels but not Cd. Also, infants exposed to Pb via human milk showed lower language trajectories than non-exposed infants.

Epidemiological research has demonstrated that child neurodevelopment is inversely related to Pb exposure, measured either in maternal blood, cord blood, or human milk (11, 20, 21), although others have reported null associations (22, 23). Comparing these studies is difficult due to variations in the sampling timing, the method and age of the neurodevelopmental tests, the number of participants, and the statistical modeling techniques. For example, the Pb concentrations in human milk in our study were lower than in the majority of previous studies in other Brazilian states, Lebanon, and Spain (24–26) and yet, it was associated with lower language skills. Although the accepted Pb levels in human milk are between 2 and 5 μg/L (27), there are no known safe commendations for blood Pb levels (28). Even blood Pb concentrations as low as 3.5 μg/dL may be linked to decreased intelligence in children, behavioral difficulties, and learning problems (29).

In a Bangladesh cohort, increased blood lead levels in children aged 20 to 40 months were associated with decreased cognitive scores (30). In Suriname, prenatal lead exposure was associated with lower receptive and expressive communication scores in children aged 1 to 2 years (31). Rural and suburban Mexican infants had a 1.5-point decrease in language development for every 1 μg/dL increase in maternal blood lead levels (32). All assessments were conducted using the Bayley-III scale. This evidence was generated in low- to middle-income countries, with the last performed in rural and suburban areas. This highlights the vulnerability of these populations to environmental pollutants exposure, aligning with our findings that socioeconomic status was a significant predictor of language development in addition to population settings.

Infants are particularly vulnerable to absorbing metals since the intestinal barrier is immature, and these elements’ permeability may be increased (33). The immaturity of other organs and systems also contributes to higher toxicity during infancy (33). The brain is more likely to be affected by Pb and neurotoxicity occurs through multiple mechanisms. Pb can cross the blood–brain barrier (BBB) via calcium channels and accumulate in astrocytes. By replacing calcium in enzyme activities, Pb can damage the mitochondria and alter lipid metabolism and decrease neurotransmitter release (34, 35). Furthermore, Pb can replace zinc in processes that regulate genetic transcription, such as zinc-finger proteins or zinc-binding sites in receptor channels. Changes in mechanisms that control gene expression during early neurodevelopment could decrease gray matter, alter myelin, and potentially lead to neurological disorders in adults (35).

Pb has been extensively investigated in relation to autism spectrum disorders (ASD) (36). A meta-analysis showed that higher levels of Pb were found in several biological samples of autistic children compared to controls (37). This suggests a possible link between the development of ASD and environmental exposure to Pb. Possible mechanisms that could explain this association include the deregulation of physiological levels of neurotransmitters (35) and the production of serum anti-ribosomal P antibodies (8). In addition, a systematic review found that infants and children exposed to Pb experience reductions in acetylcholine, glutamate and GABA levels, and NMDA receptors expression levels, which results in the decline of their reading and language abilities, increased stress response, and poor as memory (38). Pb exposure has also been associated with aggravated behavioral and immune abnormalities in autistic mouse models (39).

Pb can accumulate in bone tissue and be released into the blood and soft tissue organs in periods of physiological stress (34), such as during infants’ growth spurts, which occur typically between 5 and 12 months of life (40). Although this could explain the later effect observed in the Bayley Language Score, we should also consider improving the Bayley Scale accuracy when used in ages greater than 12 months as a possible explanation.

Our study’s limitations should be mentioned. Due to Pb quantification being lower than 50%, it had to be analyzed dichotomously (exposed or non-exposed), which may miss subtle differences in toxicity and not allow for an investigation of a dose–response relationship. Dichotomous analysis can also result in less statistical power. Also, we cannot confirm that Pb was absorbed by the infant since Pb was assessed in human milk. Regarding the outcome assessment, although recommended, the Bayley scale might not be accurate when applied as early as 12 months of infant age. Finally, while our study design could demonstrate temporality, it was not intended to establish causality.

Our results deserve appreciation because they report an estimate of high effect size, even accounting for several covariates, including maternal education and family income, infant sex, and socioeconomic status. To address the sampling biases inherent in volunteer samples, we employed pseudo-sampling weights designed to improve the representativeness of our cohort. Benefiting from longitudinal data, we computed the infant language trajectory using repeated measures rather than analyzing multiple time points in a cross-sectional manner.

In conclusion, our study shows that exposure to Pb via human milk is associated with lower language trajectory during the first 2 years of life. This emphasizes the need to revise the currently accepted levels of metals in human samples, as even low concentrations could harm vulnerable populations. More research is required to confirm the link between lead exposure and neurodevelopmental deficits in early life and to determine long-term consequences in children and adults.

## Data Availability

The original contributions presented in the study are included in the article/Supplementary material, further inquiries can be directed to the corresponding author.

## Footnotes

^1^ http://www.r-project.org
