# Supplementary material for: Lead contamination in human milk affects infants’ language trajectory: results from a prospective cohort study
Source: Front Public Health. 2024 Aug 13;12:1450570. doi: 10.3389/fpubh.2024.1450570 (PMC11347280; doi:10.3389/fpubh.2024.1450570)
Supplement: Supplementary file 1 [file Table_1.DOCX]

Supplementary Material

**Supplementary Table 1 -** Association of infants’ lead exposure and language and cognition Bayley Scales across time.

|  | Crude | | | Adjusted* | | | Adjusted╆ | | |
| --- | --- | --- | --- | --- | --- | --- | --- | --- | --- |
| **Bayley at 3 months** | Coefficient | Std. error | P-value | Coefficient | Std. Error | P-value | Coefficient | Std. Error | P-value |
| bayley_8_t1 | -1.7689 | 1.5468 | 0.255 | -1.548 | 1.536 | 0.315 | -2.467 | 1.538 | 0.110 |
| bayley_3_t1 | -1.063 | 1.844 | 0.565 | -1.031 | 1.860 | 0.580 | -0.523 | 1.8717 | 0.780 |
| **Bayley at 5-9 months** |  |  |  |  |  |  |  |  |  |
| bayley_8_t2 | -1.474 | 1.704 | 0.388 | -1.298 | 1.694 | 0.445 | -0.7396 | 1.6628 | 0.657 |
| bayley_3_t2 | -2.134 | 1.5994 | 0.184 | -2.095 | 1.587 | 0.188 | -1.731 | 1.649 | 0.295 |
| **Bayley at 10-16 months** |  |  |  |  |  |  |  |  |  |
| bayley_8_t3 | -6.24 | 2.22 | **0.006** | -6.672 | 2.254 | **0.003** | -6.66 | 2.219 | **0.003** |
| bayley_3_t3 | -2.162 | 1.927 | 0.264 | -1.934 | 1.975 | 0.329 | -2.245 | 1.886 | 0.236 |

* adjusted for family income (abep), infant age, maternal education, infant sex, breastfeeding + gestational period (weeks).
╆ adjusted for family income (abep), infant age, maternal education, infant sex, breastfeeding + gestational period (weeks) and weights.
